# Space Debris Tracking with the Poisson Labeled Multi-Bernoulli Filter

**DOI:** 10.3390/s21113684

**Published:** 2021-05-26

**Authors:** Leonardo Cament, Martin Adams, Pablo Barrios

**Affiliations:** Department of Electrical Engineering, Universidad de Chile, Av. Tupper 2007, Santiago 8370451, Chile; martin@ing.uchile.cl (M.A.); pablo.barrios@ing.uchile.cl (P.B.)

**Keywords:** random finite sets, space situational awareness, multi-target tracking, Poisson labeled multi-Bernoulli filter

## Abstract

This paper presents a Bayesian filter based solution to the Space Object (SO) tracking problem using simulated optical telescopic observations. The presented solution utilizes the Probabilistic Admissible Region (PAR) approach, which is an orbital admissible region that adheres to the assumption of independence between newborn targets and surviving SOs. These SOs obey physical energy constraints in terms of orbital semi-major axis length and eccentricity within a range of orbits of interest. In this article, Low Earth Orbit (LEO) SOs are considered. The solution also adopts the Partially Uniform Birth (PUB) intensity, which generates uniformly distributed births in the sensor field of view. The measurement update then generates a particle SO distribution. In this work, a Poisson Labeled Multi-Bernoulli (PLMB) multi-target tracking filter is proposed, using the PUB intensity model for the multi-target birth density, and a PAR for the spatial density to determine the initial orbits of SOs. Experiments are demonstrated using simulated SO trajectories created from real Two-Line Element data, with simulated measurements from twelve telescopes located in observatories, which form part of the Falcon telescope network. Optimal Sub-Pattern Assignment (OSPA) and CLEAR MOT metrics demonstrate encouraging multi-SO tracking results even under very low numbers of observations per SO pass.

## 1. Introduction

The efficient detection, tracking, and cataloging of orbiting space objects (SOs) are of paramount importance for improved Space Situational Awareness (SSA). Due to a recent collision in space and an increased number of launches, a large number of SOs now exist. As a result, the demand for modern SO tracking applications to produce more accurate and more computationally efficient detection and tracking capabilities is higher than ever.

Some of the components of the forces acting on SOs can be considered to vary in a random manner causing their orbits to change over time. Therefore, recursive Bayesian estimation methods have been adopted to detect, track, and update the states of SOs [1,2,3]. Under this paradigm, a probability density function of the multi-target state of the SOs entering the field of view (FoV) of a sensor can be propagated in time using captured observations. Subsequently, the estimated states of the SOs are propagated by updating a recursive Bayesian filter based on further observations.

Due to the large variance of various orbital parameters, limited FoVs of the sensors and typically small numbers of observations per SO pass, it is challenging to initialize new tracks and update existing tracks due to high data association uncertainty when no prior information about the SOs is available. To rectify this problem, the Admissible Region (AR) approach was proposed to limit the candidate SO orbits to be tracked by selecting either a subset of acceptable range and range rate pairs for optical observations or right ascension rate and declination rate pairs for radar observations [3]. The AR approach was further improved using additional constraints on the orbital properties such as the semi-major axis length and eccentricity and then referred to as the Constrained Admissible Region (CAR) method [3].

The hard constraints of the CAR approach have been replaced with a probabilistic representation, called the Probabilistic Admissible Region (PAR) method, to facilitate orbit initiation in Bayesian tracking methods using optical telescopic observations [4].

A popular approach used in various multi-target tracking (MTT) problems is Multiple Hypothesis Tracking (MHT) [5,6]. In recent years, Random Finite Set (RFS) algorithms based on Finite Set Statistics (FISST) [7,8] have been applied successfully to a variety of MTT problems. In particular, they estimate the cardinality as well as the multi-target state under a joint Bayesian framework and circumvent the necessity of external fragile measurement to track association algorithms.

Several Bayesian SO tracking algorithms have been developed using the CAR and PAR approaches, including the recent RFS based methods [9,10,11]. Jones et al. applied the Cardinalized Probability Hypothesis Density (CPHD) filter to the SO tracking problem, adopting the PAR and partially uniform birth (PUB) models. However, the Probability Hypothesis Density (PHD) and CPHD filters have been shown to perform less accurately than more recent labeled RFS based filters [12,13,14]. In this article, an RFS based tracking algorithm, the Poisson Labeled Multi-Bernoulli (PLMB) [12] filter, is used to process optical observations from multiple geographically disparate sensors. The PLMB filter can label tracks and has been shown to outperform the PHD and CPHD filters [13] and to perform similarly to other labeled RFS filters [12] such as the δ-Generalized Labeled Multi-Bernoulli (GLMB) or Labeled Multi-Bernoulli (LMB) filters. In contrast to the GLMB and LMB filters, however, it has the advantage of using a Poisson birth intensity which allows the direct use of the PUB model [10].

Poisson based birth filters can be used directly with the PUB-PAR concepts. Such filters include the PHD filter with complexity O(MN), where *N* is the number of target hypotheses and *M* the number of measurements. The PHD filter was superseded by the CPHD filter, the original implementation of which had complexity $O(M^{3}N)$, with a later improved implementation demonstrating a complexity of O(MN) [15].

Recently, the Poisson Multi-Bernoulli (PMB) [16] and PLMB filters were developed, in which full measurement to state assignments are estimated using the Loopy Belief Propagation (LBP) algorithm, resulting in a filter complexity of O(IMN), where *I* is the number of iterations required by the LBP algorithm to converge to a solution. The PLMB filter is an approximation of a labeled Poisson Multi-Bernoulli Mixture (PMBM) filter. While the PLMB filter propagates a single LMB distribution, the PMBM filter propagates multiple Multi-Bernoulli (MB) distributions. Its measurement to state assignment component can be solved either with Murty’s algorithm [17], giving an overall filter complexity of $O(T{\left(M+2N\right)}^{3})$ or with Gibbs sampling, under which an overall filter complexity of $O(TMN^{2})$ results [14], where *T* is the number of MB components. In [12] it was shown that the LBP-PLMB filter has a similar multi-target tracking performance to the PMBM filter, but with much higher computational efficiency. These reasons motivate the use of the PLMB filter.

The goal of this work is to build a multi-target space debris tracker based on the PLMB filter which propagates target state estimates in time using an LMB density, and a Poisson intensity to model the probability density of new born targets. Each target density will be modeled using particles due to the nonlinearity of the measurement and motion models.

The proposed methodology includes:

- The construction of a database based on published Two-Line Element (TLE) data.
- Simulated measurements based on the type of observations and locations of twelve observatories of the Falcon Telescope Network (FTN) [18].
- The use of a particle distribution for the prediction step and a Gaussian distribution for the update step. The state vector is represented in the Earth-Centered Inertial (ECI) frame of reference.
- The use of a motion model which takes into account the gravitational effect of the moon and sun and solar radiation pressure acting on the SO [19]. This is in contrast to previous work which only considered the gravitational effects of the Earth [20].
- The use of a measurement model for simulated optical measurements modelling each telescopic image.

The contributions of this article include:

- The use of a PUB-PAR-PLMB filter inspired by [10,12], for modeling the SOs initial uncertainty region within the PLMB filter. It reduces the search space for the standard birth densities, and it is more efficient and effective than a Gaussian birth density.
- The use of a PAR, which is created from a dense uniform grid, resulting in a sparse weighted particle distribution.

To place this work into perspective, in Section 2, the PAR and PUB models are explained, where the analysis and equations summarize the work of Jones et al. [10]. In Section 3, a summary of the PLMB filter prediction and update concepts are provided based on the analysis in [12]. A precise SO kinematic model is shown in Section 4, in order to account for the long periods of time which can elapse between successive measurements. A telescopic image based measurement model, which provides angular measurements of the start and end points of streaks caused by moving SOs, is given in Section 5. A method for single and multi-target state extraction from the PLMB filter posterior distribution and useful multi-target filter performance metrics are presented in Section 6. Finally, Section 7 and Section 8 show the results and conclusions, respectively.

## 2. Multi SO Initialization

Multi-SO initialization is composed of two components, namely the use of a PUB during the prediction step, and the PAR approach, which provides the single SO spatial density parameters in the update step.

Inspired by the PUB-CPHD filter in [10], a PUB-PLMB filter, combined with the PAR method for modeling the initial state of a telescopic based measurement, is adopted. The PAR is an AR which adheres to the assumption of independence between newborn and surviving targets [10]. In contrast to the PUB-CPHD filter, the proposed PUB-PLMB filter is a true multi-target tracking filter in that it estimates the identification of each target at each time step in the form of a unique label. The CPHD filter is not a true multi-target tracker, since, while it provides sequential estimates of the multi-target state, it does not provide target identity estimates, meaning that no target correlation information across time frames is provided. Furthermore, recent articles such as [12,13,14] have demonstrated superior multi-target tracking performance with labeled filters such as the PLMB filter, as opposed to non-labeled approaches such as the PHD and CPHD filters.

### 2.1. Partially Uniform Birth (PUB) Multi-Target Initiation Model

The PUB intensity generates a uniformly distributed birth density in the sensor FoV, with intensity function given by:

(1) $$D_{B}\left(x\right)=\lambda_{\beta }\cdot U\left(\theta \left(x\right);B\right)\cdot \sum_{i=1}^{J_{B}}w_{b}^{\left(i\right)}N\left(\phi \left(x\right);{\bar{\phi }}^{\left(i\right)},P_{\phi }^{\left(i\right)}\right),$$

where x is a target state in the ECI frame, θ(x) is a function that maps the target state to the observable part of the state, which, for a telescopic image, is given by $\theta \left(x\right)={\left[\alpha ,\beta ,\dot{\alpha },\dot{\beta }\right]}^{T}$. In this case, $\phi \left(x\right)={\left[s,\dot{s}\right]}^{T}$ is the non-observable part of the state, where *s* is the distance between the observer and the target, and $\dot{s}$ is the radial velocity of the target. Note that the joint vector ${\left[\theta {\left(x\right)}^{T},\phi {\left(x\right)}^{T}\right]}^{T}$ is the target state in the telescopic camera coordinate system. $\lambda_{\beta }$ is the expected number of targets of the Poisson intensity, U(θ(x);B) represents the uniform density for the observable part of the target state with boundary B given by the sensor FoV. The non-observable part of the state is modeled by a Gaussian Mixture (GM) density of $J_{B}$ components $N(\phi \left(x\right);{\bar{\phi }}^{\left(i\right)},P_{\phi }^{\left(i\right)})$, each with weight $w_{b}^{\left(i\right)}$, mean vector ${\bar{\phi }}^{\left(i\right)}$ and covariance matrix $P_{\phi }^{\left(i\right)}$.

### 2.2. Probabilistic Admissible Region (PAR) Approach for New Target Density Approximation

The PAR approach is an orbital admissible region that adheres to the assumption of independence between newborn targets and surviving SOs. These SOs obey physical energy constraints in terms of orbital semi-major axis length and eccentricity within a range of orbits of interest. In this article, Low Earth Orbit (LEO) SOs are considered.

The new SO distribution is modeled by the multiplication of the birth intensity $D_{B}\left(x\right)$ and the measurement likelihood distribution $l_{z}\left(z|x\right)$, which will be shown in detail in Section 3:

(2) $$D_{B}\left(x\right)l_{z}\left(z|x\right)\approx \lambda_{\beta }\cdot N\left(\theta \left(x\right);z,R\right)\cdot \sum_{i=1}^{J_{B}}w_{b}^{\left(i\right)}N\left(\phi \left(x\right);{\bar{\phi }}^{\left(i\right)},P_{\phi }^{\left(i\right)}\right),$$

where N(θ(x);z,R) is a Gaussian distribution on θ(x) modelling the resulting observable density for a given measurement z, with sensor noise matrix R ([10], pp. 1459).

**Figure 1:**
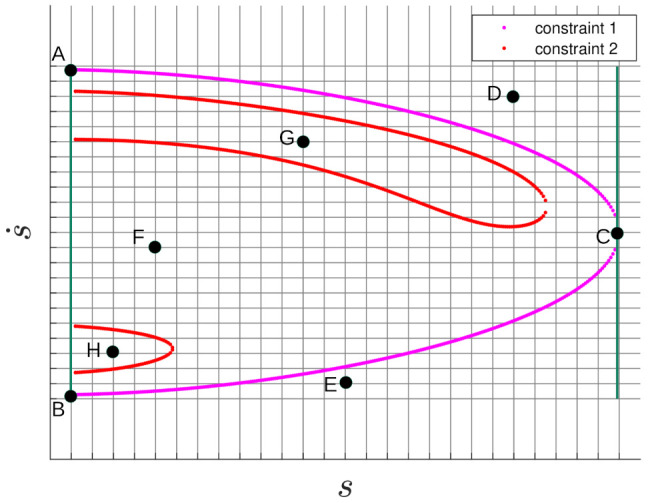
$\left(s,\;\dot{s}\right)$ constraints 1, 2 and a grid construction for satnum = 42,583.

**Figure 2:**
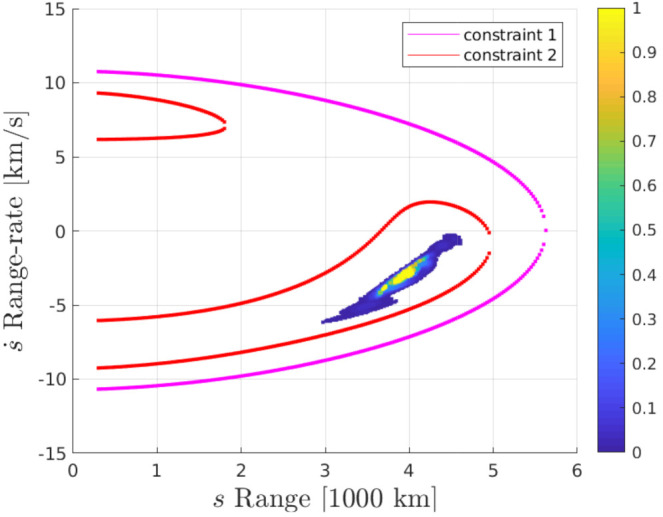
$\left(s,\;\dot{s}\right)$ PAR weighted particles for satnum = 337. Colors represent p(a,e) as indicated by the color bar. Note that in white are particles for which γ is lower than a minimum acceptable value.

In order to estimate the non-observable part of the state $\phi \left(x\right)={\left[s,\dot{s}\right]}^{T}$ based on constraints provided by the measurements, the PAR [1,10,21] methodology is used. More details about the CAR/PAR methods, including equations and their parameters, are given in Appendix A (Note that the following equations are based on the equations presented in Appendix A.). As examples, Figure 1 and Figure 2 show two curves corresponding to two constraints, namely the semi-major axis length constraint (constraint 1) and eccentricity constraint (constraint 2), calculated from simulated measurements from two randomly chosen satellites from the TLE. The constraint regions are, for constraint 1:

(3) $$c_{1}\left(\theta ,\phi ;q\right)={\dot{s}}^{2}+w_{1}\dot{s}+F\left(s\right)+\frac{\mu }{a}=0$$

and for constraint 2:

(4) $$c_{2}\left(\theta ,\phi ;q\right)=\gamma_{4}{\dot{s}}^{4}+\gamma_{3}{\dot{s}}^{3}+\gamma_{2}{\dot{s}}^{2}+\gamma_{1}\dot{s}+F\left(s\right)U\left(s\right)+\mu^{2}\left(1-e^{2}\right)=0$$

where *a* and *e* represent the semi-major axis length and eccentricity, respectively, of a hypothesized SO orbit. The Earth gravitational constant $\mu =3.986004418\times 10^{5}$ [$\frac{{\mathrm{km}}^{3}}{s^{2}}$] and $w_{1}$, F(s), U(s) and $\gamma_{1}$ to $\gamma_{4}$ are given in Appendix A. Note that these constraints are functions with variables *s* and $\dot{s}$, and form a 2D nonlinear system of equations. The solutions of the equations are given in Appendix A with their derivations given in [4,10,22].

Figure 2 shows curves corresponding to constraint 1 (magenta) and constraint 2 (red) for the SO based on simulated measurements corresponding to satnum 337 from the TLE. It also shows the posterior SO distribution, which is modeled with particles with weights represented by the color map in the figure. The particle distribution state variables and weights are determined as follows:

1. For a given measurement, z calculates the parameters of constraints 1 and 2.
2. Divide the $\left(s,\;\dot{s}\right)$ space into a grid as shown in Figure 1. To define this, it is necessary to determine the coordinates of the limiting points A, B, and C in the figure. These are determined as follows:
  (a) Obtain the points on the constraint curves 1 and 2 (corresponding to Equations (3) and (4), respectively) at the region edges, given by $a_{\max}$ = 43,000 [km] and $e_{\max}=0.4$. Note that LEO objects are defined by the constraints $a\leq a_{\max}$ and $e\leq e_{\max}$.
  (b) Find $\left(s_{right}^{c1},{\dot{s}}_{right}^{c1}\right)$, which is the coordinate at the right hand extreme of constraint 1 (Equation (3)), shown as point *C* in Figure 1. This coordinate can be found by differentiating constraint 1 with respect to $\dot{s}$ and setting $ds/d\dot{s}=0$. Note that *s* is a function of $\dot{s}$, i.e., $s=f(\dot{s})$. The solution can be determined by realizing that ${\dot{s}}_{right}^{c1}=-w_{1}/2$, then substituting for ${\dot{s}}_{right}^{c1}$ into constraint 1 (Equation (3)) and solving the resulting 6-th degree polynomial to obtain $s_{right}^{c1}$.
  (c) For $s_{\min}=300$ [km], corresponding to the lower limit of LEO SOs, obtain $\dot{s}$ (${\dot{s}}_{top}^{c1}$ and ${\dot{s}}_{bottom}^{c1}$), shown as points *A* and *B* in Figure 1:
    (5) $${\dot{s}}_{top}^{c1},{\dot{s}}_{bottom}^{c1}=-\frac{w_{1}}{2}\pm \sqrt{{\left(\frac{w_{1}}{2}\right)}^{2}-F\left(s\right)-\frac{\mu }{a_{\max}}}$$
3. For each point $\left(s,\;\dot{s}\right)$ on the grid, sample $\tilde{z}∼N\left(\cdot ;z,R\right)$ and recalculate the parameters of constraint Equations (3) and (4):
4. For each point $\left(s,\;\dot{s}\right)$ on the grid:
  (a) Solve for *a* and *e* from the constraint Equations (3) and (4):
    (6) $$a=-\frac{\mu }{{\dot{s}}^{2}+w_{1}\dot{s}+F\left(s\right)}$$
    (7) $$e=\sqrt{1+\frac{\gamma_{4}{\dot{s}}^{4}+\gamma_{3}{\dot{s}}^{3}+\gamma_{2}{\dot{s}}^{2}+\gamma_{1}\dot{s}+F\left(s\right)U\left(s\right)}{\mu^{2}}}.$$
  (b) A point on the grid is a valid LEO SO when it falls within both constrained regions, which is equivalent to $a\leq a_{\max}$ and $e\leq e_{\max}$. For example, in Figure 1, points *D* and *E* are invalid because they are outside of both constraint regions. Point *F* is within constraint 1${}^{\prime }$s region, but outside of constraint 2${}^{\prime }$s region; therefore, it is also an invalid point. However, points *G* and *H* are within both constraint regions, and are valid points. Invalid points have a weight $\tilde{\gamma }=0$, and for valid points:
  (c) evaluate the probability $\tilde{\gamma }=p\left(a,e\right)$ that the semi-major axis length and eccentricity are *a* and *e* as determined from Equations (6) and (7). The distribution p(a,e) is created using TLE data, and the procedure can be found in [10], and Appendix C.
5. The birth particle distribution ${\left\{\gamma^{j},x^{j}\right\}}_{1}^{J_{B}}$ is then given by:
  (8) $$\begin{array}{cc} \gamma^{j} & =\frac{{\tilde{\gamma }}^{j}}{\sum_{j=1}^{J_{B}}{\tilde{\gamma }}_{j}} \end{array}$$
  (9) $$\begin{array}{cc} x^{j} & =f_{t-\mathrm{radec}}^{\mathrm{eci}}\left(\left[s^{j},{\dot{s}}^{j},{\tilde{z}}^{j}\right]\right), \end{array}$$
  where $f_{t-\mathrm{radec}}^{\mathrm{eci}}\left(\cdot \right)$ is a function that maps from a topological Right Ascension and Declination (RADEC) frame to the ECI frame.

## 3. The Poisson Labeled Multi-Bernoulli (PLMB) Filter

This section describes the PLMB filter, which has been used to develop the multi object tracking algorithm. The PLMB filter is capable of modelling any number of new possible targets, and also tracking and identifying targets with a unique label. It is directly applicable to the PUB model, already demonstrated in [10] with the CPHD filter.

The multi-target tracking density is given by a LMB density characterized by its Probability Generating Functional (PGFl) $G_{X}^{\mathrm{lmb}}\left[h\right]$, ([8], p. 456), [23]:

(10) $$G_{X}^{\mathrm{lmb}}\left[h\right]=\prod_{\ell \in L}\left(1-r_{\ell }+r_{\ell }\langle f_{\ell }\left(x\right),h\left(x\right)\rangle \right),$$

where, for the RFS X, $f_{\ell }$ is the single target density of a target with label *ℓ*, $r_{\ell }$ the target’s probability of existence and h(x) a function defined in the space of the individual elements with 0≤h(x)≤1.

The birth process is modeled by a Poisson intensity $D_{B}\left(x\right)=\lambda_{B}f_{B}\left(x\right)$, where $\lambda_{B}$ is the expected number of targets to be born with spatial distribution $f_{B}\left(x\right)$. The union of Poisson and LMB densities in PGFl form is:

(11) $$\begin{array}{cc} G_{X}^{\mathrm{plmb}}\left[h\right] & =G_{B}^{p}\left[h\right]G_{Y}^{\mathrm{lmb}}\left[h\right], \end{array}$$

where $G_{B}^{p}\left[h\right]$ is a Poisson PGFl:

(12) $$G_{B}^{p}\left[h\right]=e^{D_{B}\left[h-1\right]}$$

and $G_{Y}^{\mathrm{lmb}}\left[h\right]$ is an LMB PGFl (Equation (10)). Equations (10)–(12) provide in PGFl form, the general PLMB filter equations. Their implementation will be discussed in the following subsections.

### 3.1. Multi-Target Prediction

For a prior LMB density with parameters $\left(r_{\ell },f_{\ell }\right)$, and ℓ∈L, where *L* is the set of all target labels, the prediction $\left(r_{\ell }^{\prime },f_{\ell }^{\prime }\right)$ of the LMB density is given by

(13) $$r_{\ell }^{\prime }=r_{\ell }\langle P_{S},f_{\ell }\rangle \;\mathrm{and}\;f_{\ell }^{\prime }\left(x\right)=\frac{\langle P_{S}l_{x}\left(x|\cdot \right),f_{\ell }\rangle }{\langle P_{S},f_{\ell }\rangle },$$

where $P_{S}\left(x\right)$ is the probability of survival, $l_{x}\left(x_{k}|x_{k-1}\right)$ is the kinematic model, and in general 〈ξ,η〉=∫ξ(x)η(x)dx represents the inner product. This prediction step is efficient and equivalent to the Labeled Probability Hypothesis Density (LPHD) filter prediction step in [13,23].

### 3.2. Multi-Target Update

The Bayesian update of the Poisson multi-Bernoulli PGFl (11) is a Labeled Multi-Bernoulli Mixture (LMBM) PGFl, which is approximated by an LMB PGFl. The resulting LMBM PGFl is composed of three components: New targets, detected targets, and misdetected targets [12]:

(14) $$G_{X|Z}^{+}\left[h\right]=\prod_{\ell \in L}\left(1-r_{\ell }^{+}+r_{\ell }^{+}\langle f_{\ell }^{+},h\rangle \right),$$

where $\left(r_{\ell }^{+},f_{\ell }^{+}\right)$ are the probability of existence and the probability density of the updated target identified by the label *ℓ*, respectively.

The probability of existence $r_{\ell }^{+}$ is obtained by solving the assignment problem on the cost matrix $W_{n,m}$ by applying LBP [16]. Other methods used to solve the assignment problem are Murty’s algorithm [17], and Gibbs sampling [24]. The cost matrix $W_{n,m}$ represents the weights of different combinations of targets $\ell_{n},\;n\in 1\cdots N$, for a total of *N* targets and *M* measurements $z_{m},\;m\in 1\cdots M$. Misdetected targets are represented by m=0 and n≥1 and new born targets are represented by n=0 and m≥1.

The parameters representing misdetected targets are given by:

(15) $$W_{n,0}=1-r_{\ell_{n}}^{\prime }+r_{\ell_{n}}^{\prime }\langle \left(1-P_{D}\right)f_{\ell_{n}}^{\prime },1\rangle ,$$

(16) $$r_{n,0}^{+}=\frac{r_{\ell_{n}}^{\prime }\langle \left(1-P_{D}\right)f_{\ell_{n}}^{\prime },1\rangle }{W_{n,0}},\;f_{n,0}^{+}\left(x\right)=\frac{\left(1-P_{D}\left(x\right)\right)f_{\ell_{n}}^{\prime }\left(x\right)}{\langle \left(1-P_{D}\right)f_{\ell_{n}}^{\prime },1\rangle }.$$

The parameters representing detected targets are given by:

(17) $$W_{n,m}=r_{\ell_{n}}^{\prime }\langle P_{D}l_{z}\left(z_{m}|\cdot \right)f_{\ell_{n}}^{\prime },1\rangle ,$$

(18) $$r_{n,m}^{+}=1,\;f_{\ell_{n},m}^{+}\left(x\right)=\frac{P_{D}\left(x\right)l_{z}\left(z_{m}|x\right)f_{\ell_{n}}^{\prime }\left(x\right)}{\langle P_{D}l_{z}\left(z_{m}|\cdot \right)f_{\ell_{n}}^{\prime },1\rangle },$$

and new target labels, which, in this article, are represented by a triplet of the current time step *k*, measurement index *m* and sensor index *o*, are $\ell_{N+m}=\left(k,m,o\right)$, and their parameters are given by:

(19) $$W_{0,m}=D_{K}\left(z_{m}\right)+\langle D_{B}l_{z}\left(z_{m}|\cdot \right),1\rangle ,$$

(20) $$r_{0,m}^{+}=\frac{\langle D_{B}l_{z}\left(z_{m}|\cdot \right),1\rangle }{W_{0,m}},\;f_{0,m}^{+}\left(x\right)=\frac{D_{B}\left(x\right)l_{z}\left(z_{m}|x,\ell_{N+m}\right)}{\langle D_{B}l_{z}\left(z_{m}|\cdot \right),1\rangle }.$$

The uniform distribution of clutter, $D_{K}\left(z\right)\approx \lambda_{\kappa }/V_{\kappa }$, where $\lambda_{\kappa }$ is the expected number of clutter measurements and $V_{\kappa }$ the area formed by the sensor FoV. The density $f_{0,m}^{+}\left(x\right)$ is obtained as a particle distribution by the PAR methodology described in Section 2.

The PUB intensity in Equation (1) can be expressed by separating the observed and non-observed components:

(21) $$D_{B}\left(x\right)=\lambda_{\beta }U\left(\theta \left(x\right)\right)f\left(\phi \left(x\right)\right)$$

and, therefore,

(22) $$\begin{array}{cc} \langle D_{B}\left(x\right)l_{z}\left(z_{m}|x\right),1\rangle & \approx \lambda_{\beta }\langle \frac{1}{V_{\kappa }}l_{z}\left(z_{m}|\theta \left(x\right)\right),1\rangle \langle \sum_{i=1}^{J_{B}}w_{b}^{\left(i\right)}N\left(\phi \left(x\right);{\bar{\phi }}^{\left(i\right)},P_{\phi }^{\left(i\right)}\right),1\rangle \\ \; & =\lambda_{\beta }\frac{1}{V_{\kappa }}\times 1=\frac{\lambda_{\beta }}{V_{\kappa }}. \end{array}$$

The probability of existence $r_{0,m}^{+}$ is given by:

(23) $$r_{0,m}^{+}=\frac{\lambda_{\beta }}{\lambda_{\kappa }+\lambda_{\beta }}$$

and the cost value $W_{0,m}$ is:

(24) $$W_{0,m}=\frac{\lambda_{\kappa }+\lambda_{\beta }}{V_{\kappa }}.$$

The LBP algorithm returns the weights $p_{n,m}$ that each assignment has for each label. A pseudo-code of the LBP algorithm can be found in ([16], p. 20). Then, the posterior for previously existing targets is given by:

(25) $$r_{\ell_{n}}^{+}=\sum_{m=0}^{M}p_{n,m}r_{n,m}^{+},\;f_{\ell_{n}}^{+}\left(x\right)=\sum_{m=0}^{M}p_{n,m}f_{n,m}^{+}\left(x\right),$$

and for new targets:

(26) $$r_{\ell_{N+m}}^{+}=p_{0,m}r_{0,m}^{+},\;f_{\ell_{N+m}}^{+}\left(x\right)=f_{0,m}^{+}\left(x\right).$$

Equations (25) and (26) give the parameters of the posterior multi-target distribution of Equation (14).

## 4. SO Kinematic Prediction Model

The equation of satellite motion is assumed to be [11]:

(27) $$\ddot{r}=-\frac{\mu_{E}}{{∥r∥}^{3}}r+\delta_{p}\left(r,\dot{r}\right)+a_{\epsilon },$$

where $\delta_{p}\left(r,\dot{r}\right)$ represents perturbation forces produced by different sources, and $a_{\epsilon }$ represents non-modeled forces. r and $\dot{r}$ are the position and velocity components, respectively, of the state vector x, i.e., $x={\left[r{\left(t\right)}^{T},\dot{r}{\left(t\right)}^{T}\right]}^{T}$. The predicted state is then given by:

(28) $$x_{k|k-1}=\left[\begin{array}{c} r(t_{k-1})\\ \dot{r}\left(t_{k-1}\right) \end{array}\right]+\int_{t_{k-1}}^{t_{k}}\left[\begin{array}{c} \dot{r}\left(t\right)\\ \ddot{r}\left(t\right) \end{array}\right]dt,$$

where $t_{k}$ is the time at time step *k*. Each particle of the state is propagated using the method given in [19], using the Shampine–Gordon (The Shampine–Gordon integrator is a multi-step method which uses the information from previous steps, in contrast with the Runga–Kutta method, which discards previously calculated information. Therefore, the Shampine–Gordon integrator is more efficient. The Shampine–Gordon integrator and the SO propagator C code used in this article can be found at https://www.researchgate.net/publication/340793133_High_Precision_Orbit_Propagator_C_code, (accessed on 16 May 2021). The Shampine–Gordon integrator has been used in other state propagation models including the DROMO [25], SPOOK [26,27,28], and ZUNIEM [29] propagators, in which good performance has been demonstrated.) integrator [30], which models the following forces:

- Earth central gravitation,
- Earth non-spherical forces, such as geopotential, solid tides, ocean tides,
- solar, and lunar gravitation,
- solar radiation pressure.

Further details on the implementation of the model can be found in [2,19,31,32,33].

Non-modeled perturbations $a_{\epsilon }$ can be estimated by [11,34]:

(29) $$a_{\epsilon }\left(r\left(t_{k-1}\right),\dot{r}\left(t_{k-1}\right),t,\omega \left(t_{k-1}\right)\right)=f_{\mathrm{ric}}^{\mathrm{eci}(r\left(t_{k-1}\right),\dot{r}\left(t_{k-1}\right))}\left(\left(t-t_{k-1}\right)\omega \left(t_{k-1}\right)\right)$$

where ω is a zero-mean Gaussian noise source on the second component in the object’s Radial-Intrack-Crosstrack (RIC) frame, and $f_{\mathrm{ric}}^{\mathrm{eci}}$ is the mapping that transforms a vector in the object’s RIC frame to the reference ECI frame (see Appendix B.4).

### 4.1. Particle State Prediction

For a target modeled by a particle distribution ${\left\{\gamma_{k-1},x_{k-1}\right\}}_{j=1}^{J}$, where $\gamma_{k}$ represents a particle weight at time *k*, the predicted distribution is given by:

(30) $$\begin{array}{cc} \gamma_{k|k-1} & =\gamma_{k-1} \end{array}$$

(31) $$\begin{array}{cc} x_{k|k-1} & =x_{k-1}+\int_{t_{k-1}}^{t_{k}}\left[\begin{array}{c} \dot{r}\left(t\right)\\ \ddot{r}\left(t\right) \end{array}\right]dt. \end{array}$$

### 4.2. Variable Time Step Prediction

Since, most of the time, targets are not observable, in order to reduce computational cost and improve integration performance, the target is predicted over a single time step or a long time step. It was noted in this work that using many single time step integrations is more computationally intensive than using a single long time step integration. The long time step prediction method requires the intermediate time steps and states calculated during integration and applies a spline interpolation method, function $f_{\iota }^{j}\left(\ell_{n},t\right)$ in Algorithm 1, to estimate future state values at desired time steps.

The decision rule used in the experiments presented in Algorithm 1 for using a long $\Delta T_{\iota }$ or single $t_{k}-t_{k-1}$ time step prediction is to use long prediction after $N_{th}^{\mathrm{steps}}$ single time state predictions in which the estimate is out of the FoV of all telescopes. The number of consecutive time steps where target $\ell_{n}$ is out of all telescopes’ FoVs is given by $U_{\ell_{n}}$. If the target is not observed after the long time step prediction $\Delta T_{\iota }$, another long time step prediction is executed. When the target state is within the FoV of any of the telescopes, the next predictions are carried out using the single time step prediction.
**Algorithm 1** Prediction Step Algorithm1:**Input:**$X_{k}=\left\{\left(\ell_{1},r_{1},{\left\{\gamma_{\ell_{1}}^{j},x_{\ell_{1}}^{j}\right\}}_{j=1}^{J_{\ell_{1}}}\right),\cdots ,\left(\ell_{N},r_{N},{\left\{\gamma_{\ell_{N}}^{j},x_{\ell_{N}}^{j}\right\}}_{j=1}^{J_{\ell_{N}}}\right)\right\}$,    $U_{\ell_{n}}$ ▹ Number of consecutive time steps where target $\ell_{n}$ is out all telescopes’ FoVs.2:**Output:**$X_{k}=\left\{\left(\ell_{1},r_{1},{\left\{\gamma_{\ell_{1}}^{j},x_{\ell_{1}}^{j}\right\}}_{j=1}^{J_{\ell_{1}}}\right),\cdots ,\left(\ell_{N},r_{N},{\left\{\gamma_{\ell_{N}}^{j},x_{\ell_{N}}^{j}\right\}}_{j=1}^{J_{\ell_{N}}}\right)\right\}$▹ States are overwritten.3:**for**n∈1⋯N**do**      ▹ Prediction for the distribution of each element.4:    **for** $j\in 1\cdots J_{\ell_{n}}$ **do**5:        $\gamma_{\ell_{n}}^{j}\leftarrow \gamma_{\ell_{n}}^{j}$            ▹ Weights remain the same.6:        **if** $U_{\ell_{n}}\geq N_{th}^{\mathrm{steps}}\;\&\;t_{k-1}\in \left[t_{\ell_{n}}^{\iota },t_{\ell_{n}}^{\iota }+\Delta T_{\iota }\right]$ **then**    ▹ Long term prediction.7:           $t_{\ell_{n}}^{\iota }\leftarrow t_{k-1}$8:           $f_{\iota }^{j}\left(\ell_{n},t\right)\leftarrow x_{\ell_{n}}^{j}+\int_{t_{\ell_{n}}^{\iota }}^{t_{\ell_{n}}^{\iota }+\Delta T_{\iota }}{\dot{x}}_{\ell_{n}}^{j}\left(t\right)dt$9:        **end if**10:        **if** $t_{k-1},t_{k}\in \left[t_{\ell_{n}}^{\iota },t_{\ell_{n}}^{\iota }+\Delta T_{\iota }\right]$ **then**      ▹ Interpolated prediction.11:           $x_{\ell_{n}}^{j}\leftarrow f_{\iota }^{j}\left(\ell_{n},t_{k}\right)$12:        **else**              ▹ Single time step prediction.13:           $x_{\ell_{n}}^{j}\leftarrow x_{\ell_{n}}^{j}+\int_{t_{k-1}}^{t_{k}}{\dot{x}}_{\ell_{n}}^{j}\left(t\right)dt$14:        **end if**15:    **end for**16:**end for**

## 5. SO Observation Model

**Figure 3:**
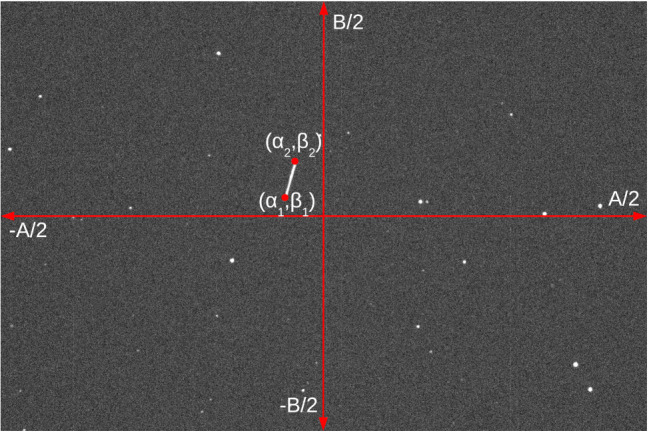
Image taken from the sidereal tracking of NORAD satellite 25,853, from the Mitre Corporation. Horizontal/vertical arrowed red lines indicate angular axes, red dots indicate the satellite coordinate $\left(\alpha_{1},\beta_{1}\right)$ at $t_{k}$ and $\left(\alpha_{2},\beta_{2}\right)$ at $t_{k}+\Delta t_{\exp}$.

An SO detection in a telescopic image is a streak. In this work, SO detection is modeled in terms of the angles $\left[\alpha_{1},\beta_{1},\alpha_{2},\beta_{2}\right]$ of the streak limits, with respect to the image center, see Figure 3. The FoV of the telescope is within the range α∈[−A/2,A/2] with respect to the horizontal axis and β∈[−B/2,B/2] with respect to the vertical axis. The measurement is not instantaneous and occurs during an elapsed time known as the *exposure time* $\Delta t_{\exp}$. The measurement can then be expressed as:

(32) $${\left[\alpha ,\beta ,\dot{\alpha },\dot{\beta }\right]}^{T}={\left[\alpha_{1},\beta_{1},\frac{\alpha_{2}-\alpha_{1}}{\Delta t_{\exp}},\frac{\beta_{2}-\beta_{1}}{\Delta t_{\exp}}\right]}^{T}.$$

An observation model for real image measurements can be found in [35]. The observation model is given by the function $\hat{z}=Hf_{\mathrm{eci}}^{\mathrm{cam}}\left(x\right)$, where $f_{\mathrm{eci}}^{\mathrm{cam}}\left(x\right)$ is the projection of the state vector into the camera spherical coordinate system and H is the observation matrix:

(33) $$H=\left[\begin{array}{cccccc} 0 & 1 & 0 & 0 & 0 & 0\\ 0 & 0 & 1 & 0 & 0 & 0\\ 0 & 0 & 0 & 0 & 1 & 0\\ 0 & 0 & 0 & 0 & 0 & 1 \end{array}\right].$$

The single target measurement model is then given by the likelihood function $l_{z}\left(z|x\right)$, modeled by the Gaussian distribution:

(34) $$l_{z}\left(z|x\right)=N\left(z;Hf_{\mathrm{eci}}^{\mathrm{cam}}\left(x\right),R\right).$$

An observed state ${\hat{z}}_{k}^{j}={\left[\alpha_{k}^{j},\beta_{k}^{j},{\dot{\alpha }}_{k}^{j},{\dot{\beta }}_{k}^{j}\right]}^{T}$ can be represented in terms of the telescope image coordinates $\left(\alpha_{1,k}^{j},\beta_{1,k}^{j},\alpha_{2,k}^{j},\beta_{2,k}^{j}\right)={\left[\alpha_{k}^{j},\beta_{k}^{j},\alpha_{k}^{j}+\Delta t_{\exp}{\dot{\alpha }}_{k}^{j},\beta_{k}^{j}+\Delta t_{\exp}{\dot{\beta }}_{k}^{j}\right]}^{T}$.

The probability of detection of a particle $x_{k|k-1}^{j}$ is given as follows:

(35) $$P_{D}\left(x_{k|k-1}^{j}\right)=\left\{\begin{array}{cc} P_{D}, & \mathrm{if}\;|\alpha_{1,k}^{j}\left|,\right|\alpha_{2,k}^{j}\left|\leq A/2\;\&\right|\beta_{1,k}^{j}\left|,\right|\beta_{2,k}^{j}|\leq B/2\\ 0, & \mathrm{otherwise}. \end{array}\right.$$

Therefore, the average probability of detection of the predicted particle distribution ${\left\{\gamma_{k|k-1}^{j},x_{k|k-1}^{j}\right\}}_{j=1}^{J}$ is

(36) $${\bar{P}}_{D}\left({\left\{\gamma_{k|k-1}^{j},x_{k|k-1}^{j}\right\}}_{j=1}^{J}\right)=\sum_{j=1}^{J}\gamma_{k|k-1}^{j}P_{D}\left(x_{k|k-1}^{j}\right).$$

The single target posterior densities are calculated based on the procedure in [11]:

1. Transform the particle state to the topocentric camera coordinates, with weights corrected by the probability of detection ${\left\{\gamma_{k|k-1}^{j},x_{k|k-1}^{j}\right\}}_{j=1}^{J}⟶\{P_{D}\left(x_{k|t-1}^{j}\right)\gamma_{k|k-1}^{j}$, $f_{\mathrm{eci}}^{\mathrm{cam}}\left(x_{k|k-1}^{j}\right){\}}_{j=1}^{J}$.
2. Approximate as a Gaussian distribution $\left(\mu_{k|t-1},P_{k|t-1}\right)$.
3. Update using a linear Kalman filter.
4. Sample the resulting distribution ${\left\{1/J,x_{k,cam}^{j}\right\}}_{j=1}^{J}$. Note that the radial components $s,\dot{s}$ do not change because they are not observed.
5. Transform from camera to ECI space $⟶{\left\{1/J,f_{\mathrm{cam}}^{\mathrm{eci}}\left(x_{t,cam}^{j}\right)\right\}}_{j=1}^{J}$.

The particle distribution of undetected targets is given by $x_{k}^{j}=x_{k|k-1}^{j}$ and:

(37) $$\gamma_{k}^{j}=\frac{\left[1-P_{D}\left(x_{k|k-1}^{j}\right)\right]\gamma_{k|k-1}^{j}}{\sum_{j^{\prime }=1}^{J}\left[1-P_{D}\left(x_{k|k-1}^{j^{\prime }}\right)\right]\gamma_{k|k-1}^{j^{\prime }}}.$$

Algorithm 2 shows the calculation of the average probability of detection, cost matrix, and state parameters representing missdetected targets under the function missdetection(·).
**Algorithm 2** Module of updated missdetected single target update.1:**Input:**$\left(\ell_{n},r_{n},{\left\{\gamma_{\ell_{n}}^{j},x_{\ell_{n}}^{j}\right\}}_{j=1}^{J_{\ell_{n}}}\right)$,2:**Output:**$W_{n,0}$, $\left(\ell_{n,0},r_{n,0},{\left\{\gamma_{\ell_{n,0}}^{j},x_{\ell_{n,0}}^{j}\right\}}_{j=1}^{J_{\ell_{n,0}}}\right)$, ${\bar{P}}_{D}^{n}$3:${\bar{P}}_{D}^{n}\leftarrow \sum_{j}\gamma_{\ell_{n}}^{j}P_{D}\left(x_{\ell_{n}}^{j}\right)$      ▹$P_{D}\left(x_{\ell_{n}}^{j}\right)$ corresponds to Equation (35).4:${\bar{P}}_{M}^{n}\leftarrow \sum_{j}\gamma_{\ell_{n}}^{j}\left(1-P_{D}\left(x_{\ell_{n}}^{j}\right)\right)$5:$W_{n,0}\leftarrow \left(1-r_{n}\right)+r_{n}{\bar{P}}_{M}^{n}$6:$\left(\ell_{n,0},r_{n,0},{\left\{\gamma_{\ell_{n,0}}^{j},x_{\ell_{n,0}}^{j}\right\}}_{j=1}^{J_{\ell_{n,0}}}\right)\leftarrow \left(\ell_{n},\frac{r_{n}{\bar{P}}_{M}^{n}}{W_{n,0}},{\left\{\gamma_{\ell_{n}}^{j}\frac{1-P_{D}\left(x_{\ell_{n}}^{j}\right)}{{\bar{P}}_{M}^{n}},x_{\ell_{n}}^{j}\right\}}_{j=1}^{J_{\ell_{n}}}\right)$

The complete pseudo code of the multi-target tracking update is given in Algorithm 3. In Algorithm 3, the following functions are used:

- particles$\mathrm{ToGM}({\left\{\gamma^{j},y^{j}\right\}}_{j=1}^{J})$: Converts a set of weighted particles to a Gaussian Mixture. This can be implemented with a variant of the Expectation Maximization (EM) algorithm, such us the Fixed Weighted-Data EM (FWD-EM) [36].
- ∼$\mathrm{GM}({\left\{w^{i},\mu^{i},P_{n}^{i}\right\}}_{i=1}^{I})$: Extracts samples from a GM distribution by first sampling the Gaussian component *i* with respect to the weights $w_{i}$, and then sampling from the normal distribution $N(\cdot ,\mu^{i},P_{n}^{i})$.
- $\mathrm{PAR}(z,o,\dot{o},R_{\mathrm{eci}}^{\mathrm{cam}}\left(t_{k},o\right))$: Calculates the weighted particle distribution of the hypothesis given by the measurement z and its observer *o* (with parameters $o,\;\dot{o},\;R_{\mathrm{eci}}^{\mathrm{cam}}\left(t_{k},o\right)$).
- $\mathrm{LBP}(W_{n,m})$: Calculates the individual contribution of each state-measurement pair from the cost matrix $W_{n,m}$ using the LBP algorithm [16].

**Algorithm 3** Update1:**Input:**$X_{k}=\left\{\left(\ell_{1},r_{1},{\left\{\gamma_{\ell_{1}}^{j},x_{\ell_{1}}^{j}\right\}}_{j=1}^{J_{\ell_{1}}}\right),\cdots ,\left(\ell_{N},r_{N},{\left\{\gamma_{\ell_{N}}^{j},x_{\ell_{N}}^{j}\right\}}_{j=1}^{J_{\ell_{N}}}\right)\right\}$,    $Z_{k}=\left\{z_{1}^{1},\cdots ,z_{M_{1}}^{1},\cdots z_{1}^{O},\cdots ,z_{M_{O}}^{O}\right\}$,2:**Output:**$X_{k}=\left\{\left(\ell_{1},r_{1},{\left\{\gamma_{\ell_{1}}^{j},x_{\ell_{1}}^{j}\right\}}_{j=1}^{J_{\ell_{1}}}\right),\cdots ,\left(\ell_{N},r_{N},{\left\{\gamma_{\ell_{N}}^{j},x_{\ell_{N}}^{j}\right\}}_{j=1}^{J_{\ell_{N}}}\right)\right\}$3:**for**o∈1⋯O**do**4:    **for** n∈1⋯N **do**5:        $W_{n,0},\;\left(\ell_{n,0},r_{n,0},{\left\{\gamma_{\ell_{n,0}}^{j},x_{\ell_{n,0}}^{j}\right\}}_{j=1}^{J_{\ell_{n,0}}}\right),\;{\bar{P}}_{D}^{n}=\mathrm{MISSDETECTION}\left(\ell_{n},r_{n},{\left\{\gamma_{\ell_{n}}^{j},x_{\ell_{n}}^{j}\right\}}_{j=1}^{J_{\ell_{n}}}\right)$6:        ${\left\{w_{n}^{i},\mu_{n}^{i},P_{n}^{i}\right\}}_{i=1}^{I_{n}}=\mathrm{PARTICLES}\mathrm{ToGM}\left({\left\{\gamma_{\ell_{n}}^{j}\frac{P_{D}\left(x_{\ell_{n}}^{j}\right)}{{\bar{P}}_{D}^{n}},f_{\mathrm{eci}}^{\mathrm{cam}}\left(x_{\ell_{n}}^{j},o\right)\right\}}_{j=1}^{J_{\ell_{n}}}\right)$7:    **end for**8:    **for** $m\in 1\cdots M_{o}$ **do**9:        **for** n∈1⋯N **do**              ▹ Detected targets.10:           $\forall i\in 1\cdots I_{n}\;q_{n,m}^{i}N\left(x;\mu_{n,m}^{i},P_{n,m}^{i}\right)\leftarrow N\left(z_{m};Hx,R_{o}\right)N\left(x;\mu_{n}^{i},P_{n}^{i}\right)$11:           $W_{n,m}\leftarrow \sum_{i}w_{n}^{i}q_{n,m}^{i}$12:           $\left(\ell_{n,m},r_{n,m},{\left\{w_{n,m}^{i},\mu_{n,m}^{i},P_{n,m}^{i}\right\}}_{i=1}^{I_{n,m}}\right)\leftarrow \left(\ell_{n},1,{\left\{\frac{w_{n}^{i}q_{n,m}^{i}}{W_{n,m}},\mu_{n,m}^{i},P_{n,m}^{i}\right\}}_{i=1}^{I_{n}}\right)$13:        **end for**14:        $W_{0,m}\leftarrow \frac{\lambda_{\beta }+\lambda_{\gamma }^{o}}{V_{\mathrm{FoV}}\left(o\right)}$              ▹ New Targets.15:        ${\left\{\gamma_{0,m}^{j},y_{0,m}^{j}\right\}}_{j=1}^{J_{0,m}}=\mathrm{PAR}\left(z_{m},o,\dot{o},R_{\mathrm{eci}}^{\mathrm{cam}}\left(t_{k},o\right)\right)$16:        $\left(\ell_{0,m},r_{0,m},{\left\{\gamma_{0,m}^{j},x_{0,m}^{j}\right\}}_{j=1}^{J_{0,m}}\right)\leftarrow \left(\left(k,m,o\right),\frac{\lambda_{\beta }}{\lambda_{\beta }+\lambda_{\gamma }^{o}}{\left\{\gamma_{0,m}^{j},f_{t-\mathrm{radec}}^{\mathrm{eci}}\left(y_{0,m}^{j}\right)\right\}}_{j=1}^{J_{0,m}}\right)$17:    **end for**18:    $p_{n,m}=\mathrm{LBP}\left(W_{n,m}\right)$              ▹ Loopy Belief Propagation.19:    **for** n∈1⋯N **do**              ▹ Updated existing targets.20:        $r_{n}\leftarrow \sum_{m=0}^{M_{o}}p_{n,m}r_{n,m}$21:        ${\left\{y_{n}^{j}\right\}}_{j=1}^{J}∼\mathrm{GM}\left({\left\{p_{n,m}r_{n,m}w_{n,m}^{i},\mu_{n,m}^{i},P_{n,m}^{i}\right\}}_{i=1,m=1}^{I_{n},M_{o}}\right)$  ▹ Sample from a GM distribution.22:        $\left(\ell_{n},r_{n},{\left\{\gamma_{\ell_{n}}^{j},x_{\ell_{n}}^{j}\right\}}_{j=1}^{J_{\ell_{n}}}\right)\leftarrow \left(\ell_{n},r_{n},{\left\{\gamma_{n,0}^{j}\frac{p_{n,0}r_{n,0}}{r_{n}},x_{n}^{j}\right\}}_{j=1}^{J_{\ell_{n}}}\right)\cup {\left\{\frac{r_{n}-p_{n,0}r_{n,0}}{Jr_{n}},f_{t-\mathrm{radec}}^{\mathrm{eci}}\left(y_{n}^{j}\right)\right\}}_{j=1}^{J}$23:    **end for**24:    **for** $m\in 1\cdots M_{o}$ **do**              ▹ Updated new targets.25:        n←N+m26:        $\left(\ell_{n},r_{n},{\left\{\gamma_{\ell_{n}}^{j},x_{\ell_{n}}^{j}\right\}}_{j=1}^{J_{n}}\right)\leftarrow \left(\ell_{0,m},p_{0,m}r_{0,m},{\left\{\gamma_{0,m}^{j},x_{0,m}^{j}\right\}}_{j=1}^{J_{0,m}}\right)$27:    **end for**28:**end for**

## 6. Multi-Target SO State Extraction and Performance Metrics

### 6.1. State Extraction

To extract the target states, the particle distribution in the ECI frame is converted to a Gaussian distribution in the RADEC frame. Usual methods to achieve this are Euclidean particle averaging. However, Euclidean averaging is not the best option for calculating the average positions of orbital trajectories. Averaging particle states converted to RADEC coordinates is also problematic due to the discontinuities that occur when $\alpha_{ra}=2\pi$ and $\beta_{dec}=\pi$. Instead, methodologies used in Riemannian manifolds are applied as follows [37,38]. Let ${\left\{\gamma^{j},x^{j}\right\}}_{j=1}^{J}$ be the particle distribution for a given target. The weighted average is calculated in spherical coordinates RADEC $x={\left[r,\alpha_{ra},\beta_{dec}\right]}^{T}$. Now, the unit vector $q_{j}$ represents the target position on the unit sphere:

(38) $$q_{j}={\left[\cos\left(\alpha_{ra}^{j}\right)\sin\left(\beta_{dec}^{j}\right)\;\sin\left(\alpha_{ra}^{j}\right)\sin\left(\beta_{dec}^{j}\right)\;\cos\left(\beta_{dec}^{j}\right)\right]}^{T}.$$

Define the logarithmic map, with respect to a reference unit vector $q_{0}$, as:

(39) $$\theta :={\mathrm{Log}}_{q_{0}}\left(q\right)=u\theta ,\;u=\frac{q-q_{0}\cos\left(\theta \right)}{\sin(\theta )},\;\theta =\arccos\left(q_{0}^{T}q\right),$$

and the exponential map as:

(40) $$q:={\mathrm{Exp}}_{q_{0}}\left(\theta \right)=ℜ\left(q_{0}\cos\left(\theta \right)+u\sin\left(\theta \right)\right),\;\theta =∥\theta ∥,\;u=\frac{\theta }{\theta }.$$

The weighted average [38] is calculated using the Gauss–Newton iterative algorithm by initiating $\bar{q}\leftarrow q_{1}$ or $\bar{q}\leftarrow q_{j^{*}}$ with $j^{*}=\arg_{j}\max{\left\{\gamma^{j}\right\}}^{J}$, and iterating until the error between two consecutive iterations is lower than a chosen threshold:

(41) $$\tau =\sum_{j=1}^{J}\gamma^{j}{\mathrm{Log}}_{\bar{q}}\left(q_{j}\right),\;\bar{q}\leftarrow {\mathrm{Exp}}_{\bar{q}}\left(\tau \right).$$

Then, for $\bar{q}={\left[{\bar{q}}_{x},{\bar{q}}_{y},{\bar{q}}_{z}\right]}^{T}$, the conversion to RADEC coordinates is given by:

(42) $${\bar{\alpha }}_{ra}=\arctan\left(\frac{{\bar{q}}_{y}}{{\bar{q}}_{x}}\right),$$

(43) $${\bar{\beta }}_{ra}=\frac{\pi }{2}-\arccos\left({\bar{q}}_{z}\right),$$

(44) $$\bar{r}=\sum_{j=1}^{J}r^{j}.$$

It is then necessary to convert back to Cartesian coordinates in the ECI frame to give $\bar{x}=f_{\mathrm{radec}}^{\mathrm{eci}}\left({\left[\bar{r},{\bar{\alpha }}_{ra},{\bar{\beta }}_{ra}\right]}^{T}\right)$.

### 6.2. Defining the Distance between Estimates and Ground Truth

To quantify the performance of the PLMB filter, distance metrics are needed. In particular, the Optimal Sub-Pattern Assignment (OSPA) and CLEAR MOT metrics used in this article require the individual error distances between estimated SO states and ground truth.

Since the estimation of SO orbital trajectories is sought in this article, it makes sense to define single SO estimate to ground-truth distance errors in terms of distances along their orbital trajectories, rather than the Euclidean distance between them. For example, if an SO is on one side of the Earth according to ground-truth, but its estimate is on the other side, the error distance should be half of the perimeter of the orbit.

When objects have different distances from the Earth ($r_{1}$ and $r_{2}$), the distance between two vectors $x_{1}$ and $x_{2}$ can be modeled by the line integral of a spiral:

(45) r(θ)=a+bθ.

Imposing $r\left(0\right)=a=\min\left(r_{1},r_{2}\right)$,

(46) $$\theta_{12}=\arccos\frac{x_{1}\cdot x_{2}}{∥x_{1}∥∥x_{2}∥},$$

where $\theta_{12}$ is the angle between $x_{1}$ and $x_{2}$, and $r\left(\theta_{12}\right)=\max\left(r_{1},r_{2}\right)$, so $b=\left(\max\left(r_{1},r_{2}\right)-\min\left(r_{1},r_{2}\right)\right)/\theta_{12}$.

The line integral is given by:

(47) $$\begin{array}{cc} \int_{0}^{\theta_{12}}r\left(\theta \right)d\theta & =\int_{0}^{\theta_{12}}a+b\theta d\theta ={\left.a\theta +b\frac{\theta^{2}}{2}\right|}_{0}^{\theta_{12}}\\ \; & =\theta_{12}\min\left(r_{1},r_{2}\right)+\frac{\max\left(r_{1},r_{2}\right)-\min\left(r_{1},r_{2}\right)}{\theta_{12}}\frac{\theta_{12}^{2}}{2}=\theta_{12}\frac{r_{1}+r_{2}}{2}, \end{array}$$

and finally:

(48) $$d_{\circ }\left(x_{1},x_{2}\right)=\theta_{12}\frac{r_{1}+r_{2}}{2}=\arccos\left(\frac{x_{1}}{∥x_{1}∥}\cdot \frac{x_{2}}{∥x_{2}∥}\right)\frac{∥x_{1}∥+∥x_{2}∥}{2}$$

is the error distance between $x_{1}$ and $x_{2}$, to be used in the multi-SO error metrics for quantifying the performance of the PLMB filter.

### 6.3. Multi-Target Tracking Metrics

The multi-SO tracking results here are compared using the OSPA [39] and OSPA${}^{\left(2\right)}$ [40] metrics and the Multi Object Tracking Precision (MOTP) and Multi Object Tracking Accuracy (MOTA) CLEAR MOT metrics [41]. Both the OSPA and OSPA${}^{\left(2\right)}$ metrics measure the precision and cardinality of two sets of targets (ground truth and estimates in this case) in one value per time step, with the difference that the OSPA${}^{\left(2\right)}$ metric is designed to evaluate labeled tracks, whereas the OSPA metric does not take labels into account. The MOTP metric gives the estimated target location errors, when correctly detected, and the MOTA metric gives the accuracy in tracking targets, taking into account missdetections, false alarms, and label switching.

## 7. Results

### 7.1. Database Construction

The database is built using simulated telescopic measurements of LEO SOs, obtained from the TLE file of 4 October 2019 (The TLE file used in the experiments, containing SO trajectory parameters from 2–6 October 2019, and a video demonstrating the resulting tracking performance can be downloaded at: https://www.dropbox.com/sh/a79hgj5hpo4vys8/AACu9OAYHLmgy4gK4V8NtqcMa?dl=0, (accessed on 16 May 2021)), which contains data related to more than 5000 SOs. The measurements are simulated by projecting the SO trajectories into the image plane of twelve telescopic cameras from the FTN. The name, location, and simulated pointing directions of the telescopes are shown in Table 1.

In the experiment, a subset of nine SOs were used. These SOs are those that produce a higher number of measurements from the telescopes when the sample frequency is seven frames per second (fps).

**Figure 4:**
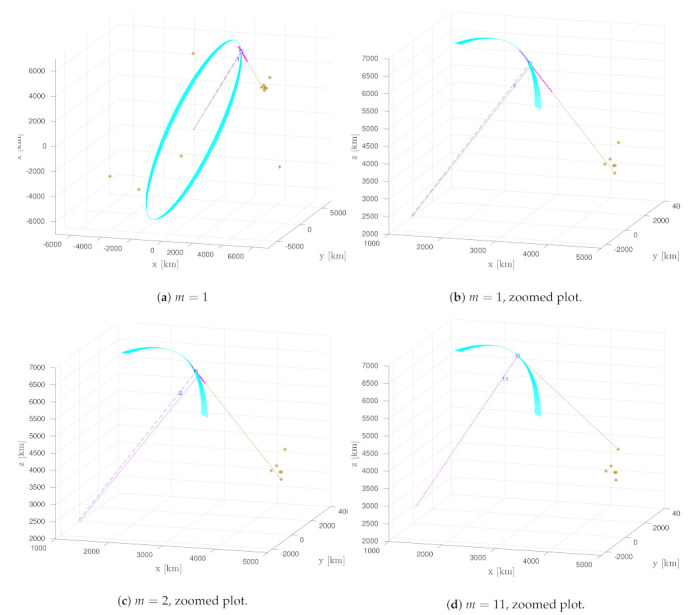
Experimental SO tracking results at three different time steps (**a**–**d**) for satnum = 337 from the TLE, where figure (**b**) is a zoomed view of the SO trajectory in figure (**a**). The complete ground truth trajectories are shown in cyan. Gold stars (*) represent the telescope positions. Circles show the ground truth SO location and their neighboring numbers *m* correspond to the *m*th measurement update. The pink points show the SO’s particles.

Experimental results at three different measurement update times (a) and (b) at m=1, (c) at m=2, (d) at m=11 are shown in Figure 4. The complete ground truth trajectories are shown in cyan. Gold stars (*) represent the telescope positions, which change over time due to the Earth’s rotation, as can be seen comparing the graphs. Circles show the ground truth SO location and their neighbouring numbers *m* correspond to the *m*th measurement update. The pink points show the SO’s particles. In Figure 4a,b, it is interesting to see the particle distribution of a new target (pink) where the high radial standard deviation ($\sigma_{r}$) of the estimate in the radial direction (gold line) with respect to the telescope’s position is evident. The error distance between ground truth and the average particle position state is 52 [km]. Figure 4c shows the update at the next time step, where it can be seen that $\sigma_{r}$ reduced significantly. Figure 4d shows the update after 11 time steps together with the measurement of satnum = 337, which in this case is observed by a different telescope with respect to Figure 4b,c, as seen by the gold line. At this time step, the error distance is greatly reduced to 0.68 [km], and $\sigma_{r}$ is reduced even further.

### 7.2. Grid Size Performance for the Probabilistic Admissible Region

The process of computing the particle distribution composed of state vectors and their associated weights can be parallelized. The PAR algorithm was implemented in C++ and processed on a computer with 16 threads. Using a uniform grid $\left(s,\dot{s}\right)$ the values p(a,e), were calculated and consequently the weighted particles $\left\{\gamma ,{\left[s,\alpha ,\beta ,\dot{s},\dot{\alpha },\dot{\beta }\right]}^{T}\right\}$ obtained. Since most of the weights γ have values close to zero, the corresponding elements are discarded, reducing the size of the particle set.

Experiments with three regular grids $\left(s,\dot{s}\right)$ were executed to demonstrate the computational times and corresponding numbers of particles in the resulting distributions:

- A 100×100 grid resulted in an average particle set size of 136±29 weighted particles and required 0.04 [s] to execute. The resulting particle set was composed of 1.36±0.29% of the total number of elements in the grid.
- A 200×200 grid resulted in an average particle set size of 465±130 weighted particles and required 0.15 [s] to execute. The resulting particle set was composed of 1.16±0.33% of the total number of elements in the grid.
- A 300×300 grid resulted in an average particle set size of 964±308 weighted particles and required 0.34 [s] to execute. The resulting particle set was composed of 1.07±0.34% of the total number of elements in the grid.

Note that, even though the number of particles generated is proportional to the number of grid cells, it is typically less than only 1.5% of this number. Furthermore, not all the $\left(s,\dot{s}\right)$ pairs in each grid cell need to be calculated. This is because only those cells which comply with the PAR constraints (i.e., only the cells enclosed within both the magenta and red regions of Figure 1, such as points G and H) are necessary.

### 7.3. Conversion of Weighted Particles to a Gaussian Mixture Distribution

The state represented by a set of weighted particles in the prediction step is converted to a GM distribution in the update step. The conversion was implemented using the FWD-EM method [36] modified using the Fuzzy C-Means method for initialization suggested in [42], under which a superior performance was demonstrated. This procedure required the FWD-EM algorithm to execute for a fixed number of Gaussian components. In this work, GMs containing between 1 and 10 components were calculated and their fit to the weighted particles compared using the Bayesian Information Criterion (BIC). In the case of a single Gaussian component, its mean and standard deviation were calculated from the weighted average and standard deviation of the particles.

In the case of multiple Gaussian components, the FWD-EM algorithm was used. Experimentally, the lowest BIC was obtained based on a single-Gaussian component in all the conversions. This possibly surprising result can be explained because the Gaussian distribution is calculated in spherical as opposed to Cartesian coordinates. For this reason, a single Gaussian component is used in the following experiments.

### 7.4. Global Tracking

Ten Monte Carlo (MC) experiments with randomly simulated observations, using the parameters in Table 2, were realized using the PLMB filter. The OSPA and CLEAR MOT metrics were computed for performance evaluation. All the parameters used in this experiment are shown in Table 2.

**Figure 5:**
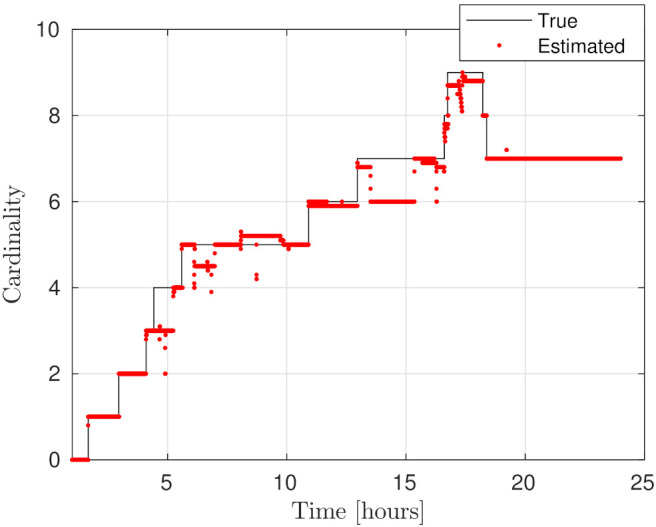
SO average cardinality for the MC experiments at each time step. The black curve shows the ground-truth and the red curve, the estimated cardinality.

**Figure 6:**
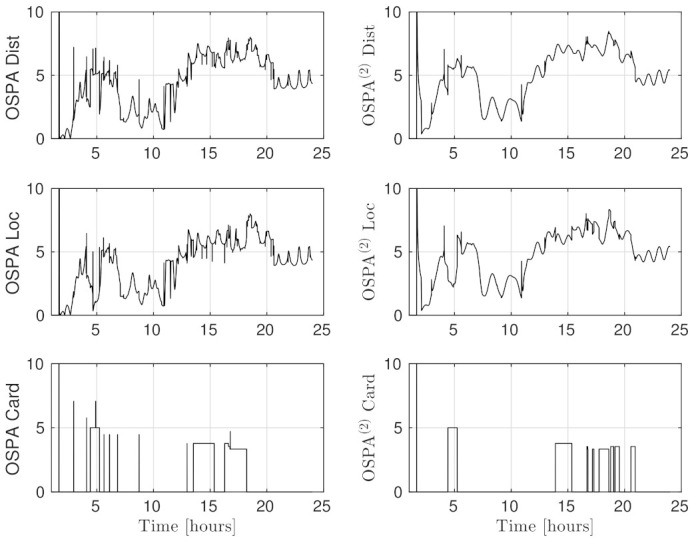
The OSPA metric values (first column) and OSPA${}^{\left(2\right)}$ metric values (second column). The first row shows the complete OSPA errors, whereas the second and third rows show the individual OSPA localization and cardinality error components. The OSPA parameters were c=10 [km], p=2 and OSPA${}^{\left(2\right)}$ window size =200 time steps equivalent to 1400 [s].

**Figure 7:**
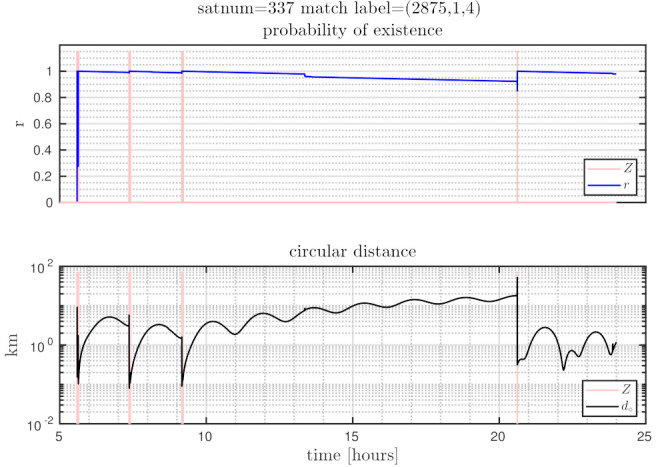
Trajectory results for satnum = 337.

**Figure 8:**
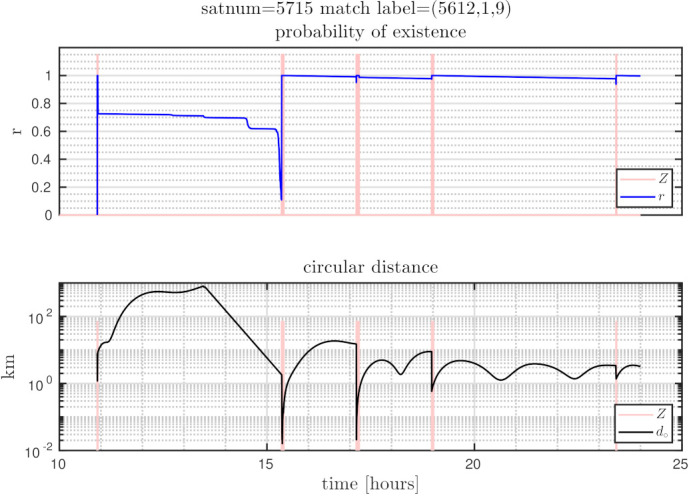
Trajectory results for satnum = 5715, first MC realization.

**Figure 9:**
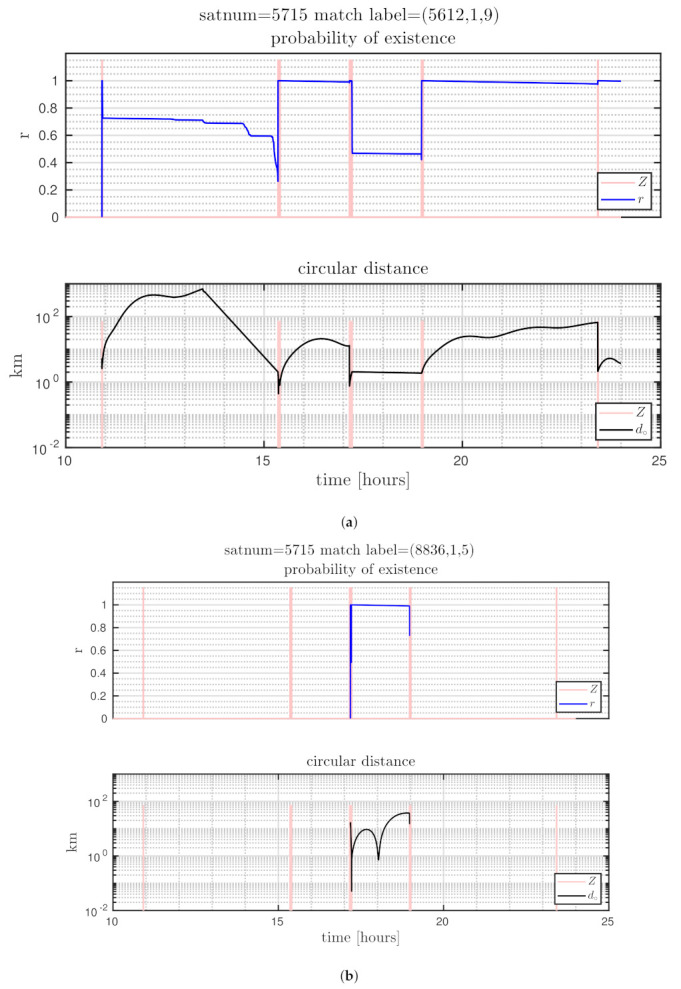
Trajectory results for satnum = 5715, second MC realization. (**a**) first hypothesis; (**b**) second hypothesis.

The distances between ground truth and estimated targets were calculated using (48). The MOTA metric in this case was 96.34±1.09% and MOTP metric was 3.14±0.24 [km]. This indicates that each target was correctly tracked more than 96% of the time since it was detected by a telescope, with a distance precision between 2.9–3.4 [km]. Figure 5 shows the resulting cardinality, in which it can be seen that most of the time the estimated number of tracks was correct, and in some intervals the error corresponds to only a single cardinality error, as can also be seen in the OSPA cardinality error, third row of Figure 6. The OSPA and OSPA${}^{\left(2\right)}$ metrics are shown in Figure 6. The first row of graphs shows the complete OSPA errors, whereas the second and third rows show the individual OSPA localization and cardinality error components. It can be seen that the OSPA distance (first row) graphs appear to be very similar to the OSPA localization graphs (second row). This is because, during all the experiments, the cardinality errors are low. The OSPA metric shows particularly low errors between 7–11 h. To explain this, it is necessary to analyze the individual tracks in Figure 7, Figure 8 and Figure 9. The first measurement of each SO occurs at different times, and its track begins at that moment. The first estimate which is modeled by the PAR produces a hypothesis with low uncertainty in the angular directions, but high uncertainty in the radial direction with respect to its observing telescope, as shown in Figure 2. This effect can be seen in Figure 8, where, during the time period 11–16 h, the radial error is very high, close to 100 [km], which is reduced to less than 1 [km] when new measurements contribute to reduce target hypothesis uncertainty. It can then be seen that the radial distance between the SO’s estimate and ground truth remains below 1 [km] for most of the time.

### 7.5. Identifying Individual SO Tracks

In Figure 7, Figure 8 and Figure 9, the first row of graphs show an SO’s estimated probability of existence *r* in blue, and the observation window time when measurements are captured in red. The second row of graphs show the ground truth to SO distance from Equation (48), which is the orbital distance used in the OSPA and CLEAR MOT metrics.

Graphs from the SO track with satnum 337 are shown in Figure 7. This SO yielded seven consecutive measurements immediately after PAR initialization. Therefore, the initial distance errors reduced significantly after this first set of seven measurements. As seen during time period 9–20 h, the SO produced no measurements, provoking an increase of distance error, but its track was successfully maintained and updated at time 20.5 h.

Figure 8 shows PLMB tracking performance, in a MC realization, for satnum 5715 in which only one measurement was obtained immediately after PAR initialization. In this case, the first graph in Figure 8 shows that r≈ 0.7 until just after 15 h, then *r* reduces before the next measurement when the probability of detection increases (The probability of detection increases above zero when the target hypothesis distribution overlaps the FoV of its observing telescope, see Equations (35) and (36)). Subsequently, *r* increases to approximately unity and remains near this value after future measurements.

Finally, Figure 9 shows the tracking result of a less successful case (another MC realization for satnum 5715) in which multiple labels, and hence target tracks, are erroneously assigned to a single SO. Figure 9a shows that a track with label (k,m,o)=(5612,1,9) is assigned to this SO. Its probability of existence *r* is significantly reduced at a time of 17 h and then increases back to unity at a time of approximately 19 h. During this time period, Figure 9b shows that a new track, with label (8836,1,5), is also assigned to satnum 5715, but with higher probability of existence *r*. This demonstrates a failure to maintain the track of satnum 5715 during this time period. Possible solutions to this problem include the implementation of another state extractor which takes into account the history of tracks with hysteresis. Other alternatives can be the fusion of tracks or the use of sets of trajectories as in [43]. Improving the state model by using a GM density, instead of a single Gaussian density as carried out here, or by using a distribution which better models the “banana shape” of the SO distribution, could also be beneficial.

### 7.6. Computational Performance

To measure the computational performance, the average time to execute ten simulations was calculated. On average, the computational time necessary for one cycle (from time *k* to k+1) of the PUB-PAR-PLMB filter was 2.5 [s]. This time consumption was made up of the following components, where all percentages are with respect to this cycle time. Note that the element Others refers to data logging, overhead, and other functions:

- Prediction: 79.94%.
  - SO particle propagation with the dynamic model (Section 4), O(N): 71.49%.
  - Spline predictor (Section 4.2), O(N): 8.31%.
  - Others: 0.14%.
- Update: 19.95%.
  - PAR algorithm (Section 2), O(M): 15.88%.
  - LBP algorithm (Section 3.2), O(NM): 0.02%.
  - $P_{D}$ (Section 5): 0.15%.
  - $f_{\mathrm{eci}}^{\mathrm{cam}}$ (Section 5): 0.30%.
  - Others: 3.60%.
- State extraction: 0.02%.
- Others: 0.09%.

It can be seen that approximately 70% of the total filter cycle time is used by the integrator within the prediction component. Note that this component forms part of the SO dynamic model and not specifically the PUB-PAR-PLMB filter. The scope of this work is not to optimize the SO propagator, but to demonstrate the suitability of the PLMB filter for multi-SO tracking. Nevertheless, it should be noted that this computational time could be reduced by using a different target predictor such as the Extended Kalman Filter (EKF) or the Unscented Kalman Filter (UKF) predictors and by using a single target state distribution which better fits the "banana shape" of SO distributions.

It can also be seen that the time required by the LBP algorithm for measurement to state assignment approximation is very small compared with the total filter cycle time. This is expected since, in these experiments, the number of measurements (*M*) and estimated targets (*N*) is low. However, since the LBP algorithm’s execution time increases with both *N* and *M*, then, as the numbers of measurements and targets increase, its execution time would start to dominate the overall filter cycle time.

## 8. Conclusions

A simulated space debris database was created based on TLE data, in which LEO satellites/debris were observed by simulated telescopes based on the FTN. A satellite measurement that corresponds to both ends of detected streaks was converted into celestial coordinates. These coordinate pairs were then used as inputs to the PLMB SO tracking algorithm. The experiment simulated measurements observed by twelve telescopes. The implementation demonstrates a particle filter version of the PLMB filter. A multi-sensor strategy was used by performing an iterative multi-target update by each sensor based on the LBP algorithm. It would be interesting to compare this with a more sophisticated multi-sensor tracking concept, for example based on Gibbs sampling, as used in [14]. The implementation of the PAR and PUB approaches for modeling the birth of the PLMB filter demonstrated a high accuracy and efficiency. The tracking results of the PLMB filter are promising and demonstrated good results even during long periods with no measurements. The performance could possibly be improved in future work with a different single target state distribution which better fits the “banana shape” of SO distributions, a more complex state extractor, by using sets of trajectories and/or another propagator/integrator.

This article has presented a proof of concept of applying the PLMB filter together with the PUB and PAR. For future work, it would be interesting to substitute various components such as the PUB, PAR, or the PLMB filter itself and compare results.

## Figures and Tables

**Table 1:** Location of the observatories of the FTN [18] with their simulated pointing directions.

Telescope Location (City, State)	Country	Longitude (East)	Latitude	Altitude (Meters)	Azimuth ${}^{\circ }$ (Simulated Pointing Direction)	0.22Elevation ${}^{\circ }$ (Simulated Pointing Direction)
Woodland Park, CO	USA	255.01	39.01	2790	179	5
Yoder, CO	USA	255.80	38.89	1961	180	5
Grand Junction, CO	USA	251.76	39.96	1380	176	5
Durango, CO	USA	252.13	37.27	1880	173	5
Sterling, CO	USA	256.80	40.65	1177	181	5
La Junta, CO	USA	256.46	37.97	1221	183	5
State College, PA	USA	282.17	40.86	317	184	5
Vicuña	Chile	289.32	−29.99	1139	4	5
Canberra	Australia	149.17	−35.29	600	260	5
Gingin	Australia	115.71	−31.36	18	230	40
Braunschweig	Germany	10.55	52.28	73	69	5
Cape Town	South Africa	18.46	−33.96	110	103	12

**Table 2:** Parameters of the experiment.

Parameter	Symbol	Value
Process noise standard deviation	ω	$10^{-7}\left[ms^{-3}\right]$
Sensor noise standard deviation	σ	2[arcsec]
Sample time	Δt	7 [s]
Exposure time	$\Delta t_{\exp}$	4 [s]
Sensor noise covariance matrix	R	$\sigma^{2}$ $\left[\begin{array}{cccc} 1 & 0 & -\Delta t_{\exp}^{-1} & 0\\ 0 & 1 & 0 & -\Delta t_{\exp}^{-1}\\ -\Delta t_{\exp}^{-1} & 0 & 2\Delta t_{\exp}^{-2} & 0\\ 0 & -\Delta t_{\exp}^{-1} & 0 & 2\Delta t_{\exp}^{-2} \end{array}\right]$
Sensor FoV for α		${\left[-2.2,2.2\right]}^{\circ }$
Sensor FoV for β		${\left[-1.2,1.2\right]}^{\circ }$
Expected number of new targets	$\lambda_{\beta }$	$8.1\times 10^{-5}$
Expected number of clutter measurements	$\lambda_{\kappa }$	0.1
Probability of detection	$P_{D}$	0.8
Probability of survival	$P_{S}$	0.99999
Maximum number of particles	*J*	100
Area to mass ratio	AMR	0.01105
PAR grid size		300×300

**Figure A1:**
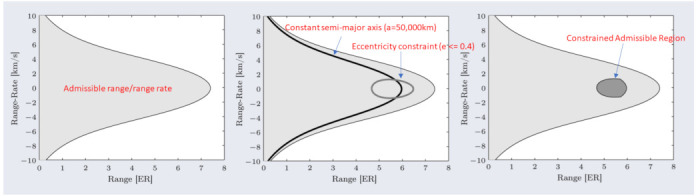
The CAR/PAR approach for initializing target tracks.

**Figure A2:**
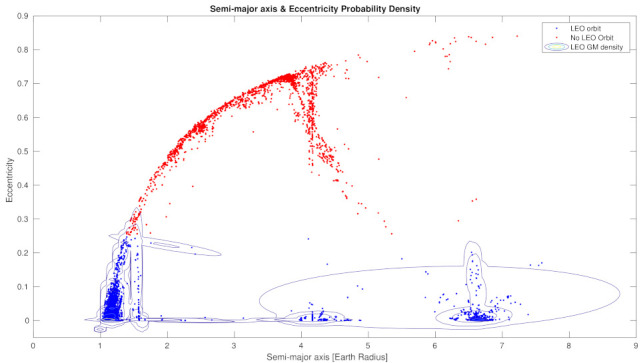
Probability density of the semi-major axis (measured in units of Earth radius) and eccentricity of LEO targets. The red points show the *non-LEO targets* and blue points the *LEO targets* of the TLE file of 2–6 October 2019. Contour lines indicate the resulting GM probability density using 30 components and a convergence tolerance of $10^{-7}$, replicated 10 times.

## Appendix A. Solution of PAR Constraint Equations

The constraint equations are derived from the celestial two-body energy equation:

(A1) $$\varepsilon =\frac{{∥\dot{r}∥}^{2}}{2}-\frac{\mu }{∥r∥}$$

with energy ε, where μ is the gravitational constant of the Earth, r is the position, and $\dot{r}$ is the velocity of the state vector in the ECI frame. The concept of these constraints is shown in Figure A1.

The measurement is assumed to take place in a topological RADEC coordinate system. This is a spherical coordinate system parallel to the ECI frame. The relation between the measurement and the state is given by [10]:

(A2) $$r=o+su_{s}$$

(A3) $$\dot{r}=\dot{o}+\dot{s}u_{s}+s\left(\dot{\alpha }u_{\alpha }+\dot{\beta }u_{\beta }\right)$$

where the Cartesian axes of the measurement are:

- $u_{s}={\left[\cos\left(\alpha \right)\cos\left(\beta \right)\;\;\;\;\;\sin\left(\alpha \right)\cos\left(\beta \right)\;\;\;\;\;\sin\left(\beta \right)\right]}^{T}$
- $u_{\alpha }={\left[-\sin\left(\alpha \right)\cos\left(\beta \right)\;\;\cos\left(\alpha \right)\cos\left(\beta \right)\;\;\;\;\;\;0\right]}^{T}$
- $u_{\beta }={\left[-\cos\left(\alpha \right)\sin\left(\beta \right)\;\;-\sin\left(\alpha \right)\sin\left(\beta \right)\;\;\cos\left(\beta \right)\right]}^{T}$,

$s=su_{s}$ is the position vector with respect to the observer and s=∥s∥. o is the center of the observer respect to the ECI frame, and $\dot{o}$ is its velocity. The observer velocity $\dot{o}=\omega_{⊕}\times o$, where $\omega_{⊕}$ is the angular rotation of the Earth in the ECI frame, the calculation of which is given in Appendix B.

### Appendix A.1. Constraint 1

The semi-major axis length constraint is provided by the energy:

(A4) $$\varepsilon =-\frac{\mu }{2a}$$

where substituting (A2) and (A3) into (A1), for $a\leq a_{\max}$, results in:

(A5) $$c_{1}\left(\theta ,\phi ;q\right)={\dot{s}}^{2}+w_{1}\dot{s}+F\left(s\right)+\frac{\mu }{a_{\max}}\leq 0$$

where

(A6) $$F\left(s\right)=w_{2}s^{2}+w_{3}s+w_{4}-\frac{2\mu }{\sqrt{s^{2}+w_{5}s+w_{0}}}$$

with:

(A7) $$\begin{array}{cc} w_{0} & ={∥o∥}^{2},\\ w_{1} & =2\dot{o}\cdot u_{s},\\ w_{2} & ={\dot{\alpha }}^{2}\cos^{2}\left(\beta \right)+{\dot{\beta }}^{2}\\ w_{3} & =2\dot{\alpha }\left(\dot{o}\cdot u_{\alpha }\right)+2\dot{\beta }\left(\dot{o}\cdot u_{\beta }\right),\\ w_{4} & ={∥\dot{o}∥}^{2},\\ w_{5} & =2o\cdot u_{s} \end{array}$$

### Appendix A.2. Constraint 2

The eccentricity constraint is given by the angular momentum $h=r\times \dot{r}$, where the energy is:

(A8) $$\varepsilon =-\frac{\mu^{2}\left(1-e^{2}\right)}{2{∥h∥}^{2}}$$

and substituting (A2) and (A3) into (A1), for $e\leq e_{\max}$ results in:

(A9) $$c_{2}\left(\theta ,\phi ;q\right)=\gamma_{4}{\dot{s}}^{4}+\gamma_{3}{\dot{s}}^{3}+\gamma_{2}{\dot{s}}^{2}+\gamma_{1}\dot{s}+\gamma_{0}\leq 0$$

where

(A10) $$\begin{array}{cc} \gamma_{0} & =F\left(s\right)U\left(s\right)+\mu^{2}\left(1-e_{\max}^{2}\right)\\ \gamma_{1} & =F\left(s\right)P\left(s\right)+w_{1}U\left(s\right)\\ \gamma_{2} & =U\left(s\right)+c_{0}F\left(s\right)+w_{1}P\left(s\right)\\ \gamma_{3} & =P\left(s\right)+c_{0}w_{1}\\ \gamma_{4} & =c_{0} \end{array}$$

where the coefficients $c_{i}$ are calculated from the angular momentum:

(A11) $$h=\dot{s}h_{1}+s^{2}h_{2}+sh_{3}+h_{4}$$

and:

(A12) $$\begin{array}{cc} P(s) & =c_{1}s^{2}+c_{2}s+c_{3} \end{array}$$

(A13) $$\begin{array}{cc} U(s) & =c_{4}s^{4}+c_{5}s^{3}+c_{6}s^{2}+c_{7}s+c_{8} \end{array}$$

with:

(A14) $$\begin{array}{cc} c_{0} & ={∥h_{1}∥}^{2}\\ c_{1} & =2h_{1}\cdot h_{2}\\ c_{2} & =2h_{1}\cdot h_{3}\\ c_{3} & =2h_{1}\cdot h_{4}\\ c_{4} & ={∥h_{2}∥}^{2}\\ c_{5} & =2h_{2}\cdot h_{3}\\ c_{6} & =2h_{2}\cdot h_{4}+{∥h_{3}∥}^{2}\\ c_{7} & =2h_{3}\cdot h_{4}\\ c_{8} & ={∥h_{4}∥}^{2} \end{array}$$

and

(A15) $$\begin{array}{cc} h_{1} & =o\times u_{s}\\ h_{2} & =u_{s}\times \left(\dot{\alpha }u_{\alpha }+\dot{\beta }u_{\beta }\right)\\ h_{3} & =u_{s}\times \dot{o}+o\times \left(\dot{\alpha }u_{\alpha }+\dot{\beta }u_{\beta }\right)\\ h_{4} & =o\times \dot{o}. \end{array}$$

## Appendix B. Coordinate Transformations

The variables, regarding Earth orbit variations, used in the coordinate transformations, on 4 October 2019 (the date of our experiment) were:

- $t_{lod}=--0.0003216\left[s\right]$
- $x_{p}=0.195512^{\prime \prime }=9.478689242\times 10^{-7}$ [rad].
- $y_{p}=0.308733^{\prime \prime }=1.496779822\times 10^{-6}$ [rad].
- UTC1-UTC: dutc1=−0.1536855 [s].
- $ddpsi=--0.116622^{\prime \prime }=--5.65399411183563\times 10^{-7}$ [rad].
- $ddeps=--0.009989^{\prime \prime }=--4.84280386060315\times 10^{-8}$ [rad].
- TAI-UTC: dat=37 [s].

### Appendix B.1. Observer’s Position and Velocity

The Earth’s angular velocity is $\omega_{E}=7.29211514670698\times 10^{-5}\times \left(1-\frac{t_{lod}}{86400}\right)$ and, as a vector in Earth-Centered, Earth-Fixed (ECEF) frame, it is $\omega_{E}={\left[0,0,\omega_{E}\right]}^{T}$. The Earth’s angular rotation in the ECI frame is $\omega_{⊕}=R_{\mathrm{pns}}\omega_{E}$, where $R_{\mathrm{pns}}=R_{\mathrm{prec}}R_{\mathrm{nute}}R_{\mathrm{st}}$ are given in Vallado’s [44] MATLAB functions (*precess.m*, *nutation.m* and *sidereal.m*). Note that $R_{\mathrm{pns}}$ is time dependent; therefore, it must be calculated at each time step.

The observer position in the ECI frame is given by $o=R_{\mathrm{pns}}P_{M}o_{ECEF}$, where $P_{M}$ and $o_{ECEF}$ are calculated with Equations (A16) and (A17), respectively.

The observer velocity in the ECI frame is given by $\dot{o}=\omega_{⊕}\times o$.

### Appendix B.2. Polar Motion Matrix

The polar motion matrix is:

(A16) $$P_{M}\left(x_{p},y_{p}\right)=\left[\begin{array}{ccc} \cos x_{p} & 0 & -\sin x_{p}\\ \sin x_{p}\sin y_{p} & \cos y_{p} & \cos x_{p}\sin y_{p}\\ \sin x_{p}\cos y_{p} & -\sin y_{p} & \cos x_{p}\cos y_{p} \end{array}\right]\approx \left[\begin{array}{ccc} 1 & 0 & -x_{p}\\ 0 & 1 & y_{p}\\ x_{p} & -y_{p} & 1 \end{array}\right].$$

### Appendix B.3. Geodetic Coordinate to ECEF Frame

$o_{ECEF}=f_{\mathrm{geo}}^{\mathrm{ecef}}\left(o_{\mathrm{geo}}\right)$, where $o_{\mathrm{geo}}$ is the observer position in latitude λ, longitude $\phi_{gc}$ and altitude $h_{obs}$.

(A17) $$o_{ECEF}=\left[\begin{array}{c} \left(N+h_{obs}\right)\cdot \cos\left(\lambda \right)\cdot \cos\left(\phi_{gc}\right)\\ \left(N+h_{obs}\right)\cdot \cos\left(\lambda \right)\cdot \sin\left(\phi_{gc}\right)\\ \left(\left(1-e_{⊕}^{2}\right)\cdot N+h_{obs}\right)\cdot \sin\left(\lambda \right) \end{array}\right],$$

where

- the radius of curvature at prime meridian $N=\frac{R_{⊕}}{\sqrt{1-e_{⊕}^{2}\sin^{2}\left(\lambda \right)}}$,
- the equatorial Earth radius $R_{⊕}=6378.137$ [km],
- the Earth’s eccentricity $e_{⊕}=8.1819190842622\times 10^{-2}$.

### Appendix B.4. RIC to ECI Frame Transform

For a target $x={\left[r^{T},\dot{r^{T}}\right]}^{T}$, the mapping function from the RIC to the ECI frame is given by:

(A18) $$f_{\mathrm{ric}}^{\mathrm{eci}}\left(y\right)=\left[u\;v\;w\right]y,$$

where

(A19) $$\begin{array}{cccccccc} u & =\frac{r}{∥r∥}, & \; & w & =\frac{r\times \dot{r}}{∥r\times \dot{r}∥}, & \; & v & =\frac{w\times u}{∥w\times u∥}. \end{array}$$

### Appendix B.5. Topological RADEC to ECI Frame Transform

For a telescope with coordinates o, $\dot{o}$, the transformation from the topological RADEC to the ECI frame $x=f_{t-\mathrm{radec}}^{\mathrm{eci}}\left(z\right)$, where $z=[r,\alpha ,\beta ,\dot{r},\dot{\alpha },\dot{\beta }]$, is given by

(A20) $$x=\left[\begin{array}{c} r\cos\left(\beta \right)\cos\left(\alpha \right)\\ r\cos\left(\beta \right)\sin\left(\alpha \right)\\ r\sin\left(\beta \right)\\ \dot{r}\cos\left(\beta \right)\cos\left(\alpha \right)-z\cos\left(\alpha \right)\dot{\beta }-y\dot{\alpha }\\ \dot{r}\cos\left(\beta \right)\sin\left(\alpha \right)-z\sin\left(\alpha \right)\dot{\beta }+x\dot{\alpha }\\ \dot{r}\sin\left(\beta \right)+r\dot{\beta }\cos\left(\beta \right) \end{array}\right]+\left[\begin{array}{c} o\\ \dot{o} \end{array}\right].$$

### Appendix B.6. ECI to Telescope Camera Coordinate Transformation

It is first necessary to transform from the ECI coordinates x to Cartesian camera coordinates $x_{\mathrm{cam}}$, and then to spherical camera coordinates $z_{\mathrm{cam}}$:

(A21) $$x_{\mathrm{cam}}=\left[\begin{array}{c} R_{\mathrm{eci}}^{\mathrm{cam}}\left(t\right)\left(r-o\right)\\ R_{\mathrm{eci}}^{\mathrm{cam}}\left(t\right)\left(\dot{r}-\dot{o}\right) \end{array}\right]$$

where $R_{\mathrm{eci}}^{\mathrm{cam}}\left(t\right)=R_{\mathrm{eci}}^{\mathrm{sez}}\left(t\right)R_{\mathrm{sez}}^{\mathrm{cam}}$, $R_{\mathrm{eci}}^{\mathrm{sez}}\left(t\right)$ is the rotation matrix from the ECI to the South-East-Zenith (SEZ) frame [44] at time *t*, and $R_{\mathrm{sez}}^{\mathrm{cam}}$ (Equation (A28)) is the rotation matrix of a telescope pointing in direction $\left(\alpha_{c},\beta_{c}\right)$, with respect to the Earth plane.

Let $x_{\mathrm{cam}}={\left[x,y,z,\dot{x},\dot{y},\dot{z}\right]}^{T}$, and let $s=\sqrt{x^{2}+y^{2}}$; then,

(A22) $$\begin{array}{cc} r & =\sqrt{s^{2}+z^{2}} \end{array}$$

(A23) $$\alpha =\{\begin{array}{ll} \arctan\left(\frac{y}{-x}\right) & \mathrm{if}\;s>\epsilon \\ \arctan\left(\frac{\dot{y}}{-\dot{x}}\right) & \mathrm{otherwise} \end{array}$$

(A24) $$\beta =\{\begin{array}{ll} \arctan\left(\frac{z}{s}\right) & \mathrm{if}\;s>\epsilon \\ \mathrm{sign}\left(z\right)\frac{\pi }{2} & \mathrm{otherwise} \end{array}$$

(A25) $$\dot{r}=\frac{x\dot{x}+y\dot{y}+z\dot{z}}{r}$$

(A26) $$\dot{\alpha }=\{\begin{array}{ll} \frac{y\dot{x}-x\dot{y}}{s^{2}} & \mathrm{if}\;s>\epsilon \\ 0 & \mathrm{otherwise} \end{array}$$

(A27) $$\dot{\beta }=\{\begin{array}{ll} \frac{{\dot{z}s}^{2}-z(x\dot{x}+y\dot{y})}{r^{2}s} & \mathrm{if}\;s>\epsilon \\ 0 & \mathrm{otherwise} \end{array}$$

where ϵ is a threshold representing a value close to 0, since $\dot{\alpha }$ and $\dot{\beta }$ are undefined when s=0. Here, $\epsilon =10^{-10}$, and

(A28) $$R_{\mathrm{sez}}^{\mathrm{cam}}=\left[\begin{array}{ccc} \cos\alpha_{c}\cos\beta_{c} & -\sin\alpha_{c}\cos\beta_{c} & -\sin\beta_{c}\\ \sin\alpha_{c} & \cos\alpha_{c} & 0\\ \cos\alpha_{c}\sin\beta_{c} & -\sin\alpha_{c}\sin\beta_{c} & \cos\beta_{c} \end{array}\right]$$

## Appendix C. Construction of Semi-Major Axis Length and Eccentricity GM Distribution

The construction of the p(a,e) density is realized with the procedure described in [10]. The density $p\left(q_{s}\right)=p\left(a,e\right)$ is a GM approximation of the *a*-*e* density derived via the public TLE catalog from 4 October 2019. Using the 1118 samples consistent with the *a* and *e* constraints in ([10], Table I), the EM algorithm with a convergence tolerance of $10^{-7}$ is used to generate a GM with 30 components. Figure A2 shows the *a* and *e* values for more than 10,000 SOs from the TLE and the resulting GM distribution.

The TLE provides the orbit eccentricity but does not directly provide the semi-major axis length. Instead, it provides the the mean motion *n*, which is the number of revolutions that the satellite moves around the Earth in one day. Using the Kepler equation $n^{2}a^{3}=\mu$, it is possible to obtain the semi-major axis length *a* in terms of the mean motion *n*.
